# 2287 ECG and echo characteristics in familial partial lipodystrophy: The impact of Lamin A variants

**DOI:** 10.1017/cts.2018.164

**Published:** 2018-11-21

**Authors:** Abdelwahab J. Eldin, Rasimcan Meral, Adam H. Neidert, Diana Rus, Rita Hench, Hakan Oral, Elif A. Oral

**Affiliations:** University of Michigan School of Medicine

## Abstract

OBJECTIVES/SPECIFIC AIMS: Familial partial lipodystrophy (FPLD) is an inherited, rare syndrome characterized by selective absence of adipose tissue from extremities which is associated with severe insulin resistance, and metabolic dyslipidemia (with hypertriglyceridemia, and low HDL) Typically, 30%–50% of patients with FPLD demonstrate a pathogenic variant in Lamin A (LMNA) gene that is associated with inherited cardiomyopathy and arrhythmia syndromes. We inquired the prevalence of having abnormal ECGs and echocardiograms in FPLD and whether there is a difference in evaluated parameters with respect to genotype. METHODS/STUDY POPULATION: We conducted a retrospective review of an established a cohort of 58 patients (age range: 12–71, M/F 8/50) with FPLD. Demographic characteristics, genotype, fasting triglyceride, hemoglobin A1c, LDL, and HDL levels were collected; ECGs and echocardiograms were also interrogated. RESULTS/ANTICIPATED RESULTS: Out of 58 patients, 22 (38 %) displayed a pathogenic variant in the LMNA gene. In total, 71% of patients (41/58) had an abnormal ECG and echocardiogram; 40% (23/58) of the patients displayed an arrhythmia on the ECGs (13 in the patients with LMNA variants and 10 in the non-LMNA group). The likelihood of having an arrhythmia was significantly higher in the patients with LMNA variants versus those without (odds ratio of 3.4, CI: 1.1–10.6). DISCUSSION/SIGNIFICANCE OF IMPACT: The overall prevalence of abnormal ECHO and/or ECG is high at 45/58 (78 %) in FPLD. Patients with LMNA variants have a 3.4 times increased risk of developing cardiac arrhythmias compared to those without. We recommend vigilant, monitoring for cardiac disease in FPLD and for arrhythmias in patients with FPLD and LMNA variants.

